# Defective CFTR- β-catenin interaction promotes NF-κB nuclear translocation and intestinal inflammation in cystic fibrosis

**DOI:** 10.18632/oncotarget.11747

**Published:** 2016-08-31

**Authors:** Kaisheng Liu, Xiaohu Zhang, Jie Ting Zhang, Lai Ling Tsang, Xiaohua Jiang, Hsiao Chang Chan

**Affiliations:** ^1^ Epithelial Cell Biology Research Center, Key Laboratory for Regenerative Medicine of the Ministry of Education of China, School of Biomedical Sciences, Faculty of Medicine, The Chinese University of Hong Kong, Hong Kong SAR, PR China; ^2^ School of Biomedical Sciences Core Laboratory, Shenzhen Research Institute, The Chinese University of Hong Kong, Shenzhen, PR China; ^3^ Sichuan University-The Chinese University of Hong Kong Joint Laboratory for Reproductive Medicine, West China Second University Hospital, Chengdu, PR China

**Keywords:** CFTR, β-catenin, NF-κB, small intestine, inflammation

## Abstract

While inflammation with aberrant activation of NF-κB pathway is a hallmark of cystic fibrosis (CF), the molecular mechanisms underlying the link between CFTR defect and activation of NF-κB-mediated pro-inflammatory response remain elusive. Here, we investigated the link between CFTR defect and NF-κB activation in ΔF508*cftr*^−/−^ mouse intestine and human intestinal epithelial cell lines. Our results show that the NF-κB/COX-2/PGE_2_ pathway is activated whereas the β-catenin pathway is suppressed in CF mouse intestine and CFTR-knockdown cells. Activation of β-catenin pathway by GSK3 inhibitors suppresses CFTR mutation/knockdown-induced NF-κB/COX-2/PGE_2_ pathway in ΔF508 mouse intestine and CFTR-knockdown cells. In contrast, suppression of β-catenin signaling induces the nuclear translocation of NF-κB. In addition, CFTR co-localizes and interacts with β-catenin while CFTR mutation disrupts the interaction between NF-κB and β-catenin in mouse intestine. Treatment with proteasome inhibitor MG132 completely reverses the reduced expression of β-catenin in Caco-2 cells. Collectively, these results indicate that CFTR stabilizes β-catenin and prevents its degradation, defect of which results in the activation of NF-κB-mediated inflammatory cascade. The present study has demonstrated a previously unsuspected interaction between CFTR and β-catenin that regulates NF-κB nuclear translocation in mouse intestine. Therefore, our study provides novel insights into the physiological function of CFTR and pathogenesis of CF-related diseases in addition to the NF-κB-mediated intestinal inflammation seen in CF.

## INTRODUCTION

Cystic fibrosis (CF) is an autosomal recessive genetic disease mostly occurred in Caucasian [1]. The devastating disease is caused by mutations in the cystic fibrosis transmembrane conductance regulator (*CFTR*) gene which encodes a cAMP-regulated anion channel at the apical membrane of epithelial cells in various organs [2]. Though more than 2000 mutations have been identified in CF patients (http://www.genet.sickkids.on.ca/cftr/app), deletion of the phenylalanine at position 508 (ΔF508) is the most frequent mutation occurring in over 80% of CF patients [3, 4]. *CFTR* mutations lead to a wide spectrum of clinical manifestations including pancreatic insufficiency, focal biliary cirrhosis, infertility, and chronic airway obstructions that destroy the lung [5]. While it was long held that progressive lung disease characterized by chronic inflammation is the prime cause of morbidity in CF, inflammatory manifestation has also been observed in other tissues and organs including gastrointestinal tract [6–11]. For instance, infiltration of mast cells and neutrophils, as well as upregulation of inflammation-associated cytokines have been observed in CF mice and patients, suggesting augmented intestinal epithelial permeability and inflammation [7, 8]. However, the exact mechanisms underlying the intestinal inflammation in CF remain elusive.

The inherent inflammation in CF lung diseases has been associated with aberrantly-activated NF-κB-mediated inflammatory responses [12, 13]. This notion is supported by a large body of evidence showing increased activation of NF-κB and subsequent excessive pro-inflammatory cytokines in CF cell lines where infection is not an issue [14–16]. In addition, increased levels of inflammatory cytokines and mediators, such as interleukins, tumor necrosis factor-α (TNF-α) and prostaglandin E_2_ (PGE_2_) have been detected in the sputum and bronchoalveolar lavage fluid (BALF) of CF patients [17–19]. Our previous studies have demonstrated that CFTR functions as a negative regulator of COX-2/PGE_2_–mediated pro-inflammatory response in airway and prostate epithelial cells, defective of which results in excessive activation of NF-κB and over production of PGE_2_ [20–22]. Collectively, these findings point toward a scenario that defective CFTR leads to exaggerated NF-κB-mediated pro-inflammatory responses that are not related to bacterial infection. However, how this NF-κB-mediated pro-inflammatory signaling is activated in CF is unknown.

The Wnt/β-catenin signaling cascade is implicated in the control of stem cell activity, cell proliferation, and cell survival of the gastrointestinal epithelium. Interestingly, β-catenin has been shown to physically interact with NF-κB in the cytoplasm, which leads to the reduction of NF-κB nuclear translocation and transcriptional activation in intestinal epithelial cells and cancers cells [23, 24]. Furthermore, anti-inflammatory role of Wnt/β-catenin pathway has been revealed in intestinal epithelial cells in response to bacterial infection recently [24–26]. Given the reported involvement of NF-κB in regulating inflammatory responses in the CF airways and other tissues, we hypothesize that CFTR regulates NF-κB activity through β-catenin pathway, dysfunction of which may lead to aberrant activation of NF-κB/COX-2/PGE_2_ cascade and exaggerated inflammatory response observed in CF intestine. We undertook the present study to test this hypothesis and focused on the link between CFTR and NF-κB.

## RESULTS

### ΔF508 mutation leads to intestinal inflammation in mice

**Figure 1:**
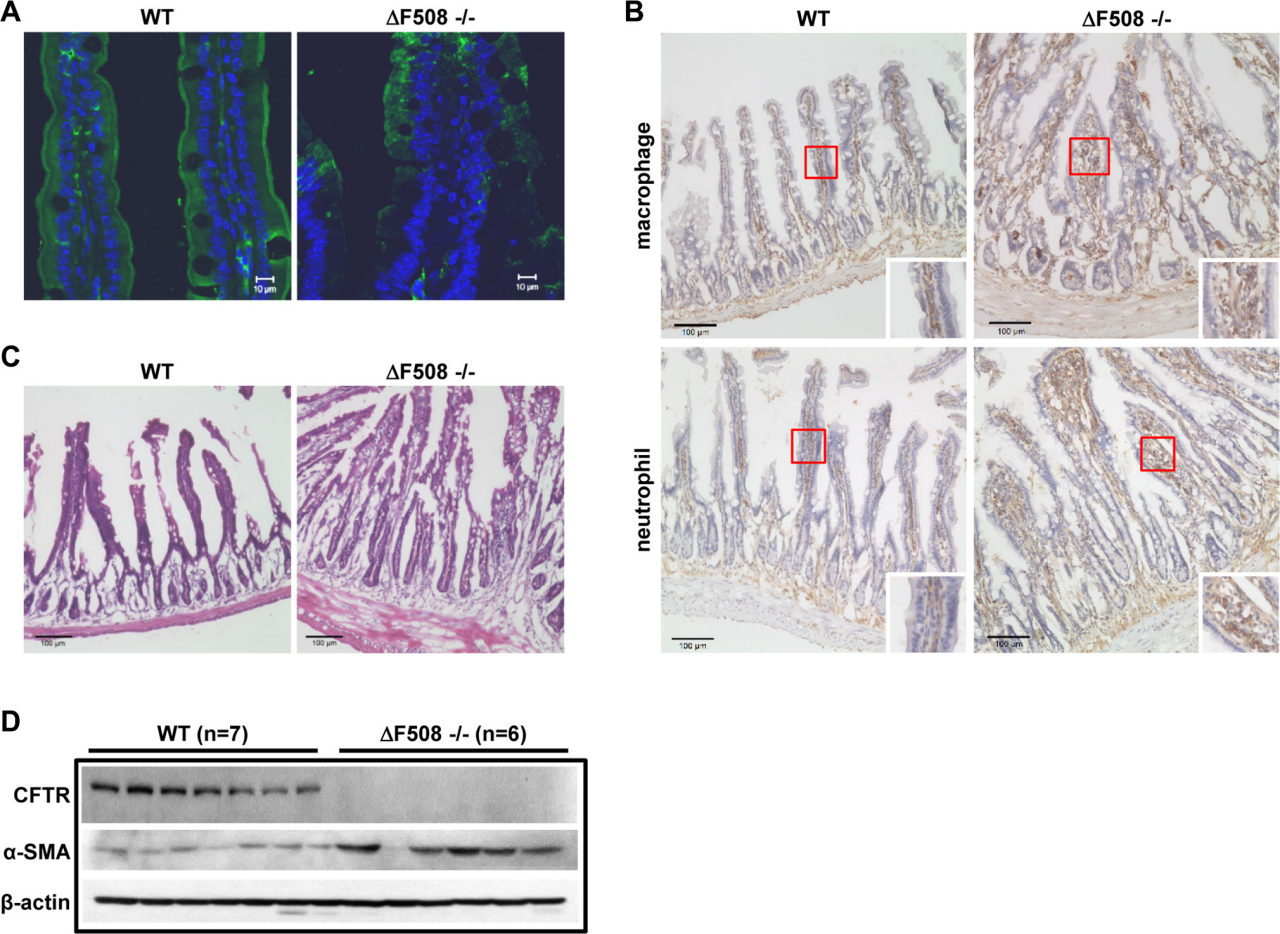
ΔF508 mutation leads to intestinal inflammation in mice (**A**) Immunofluorescence staining of CFTR (Anti-CFTR, 1:20, ACL-006, Alomone Labs.) in WT and ΔF508*cftr*^−/−^ mouse small intestine, scale bar = 10 μM, (**B**) Immunohistochemistry staining of macrophages and neutrophils (Rat anti-mouse F4/80, 1:50, Bio-Rad, Rat anti-mouse Ly-6B.2 Alloantigen, 1:50, Bio-Rad) in WT and ΔF508*cftr*^−/−^ small intestine. Scale bar: 100 μm. (**C**) H&E staining shows significant increase of the thickness of smooth muscle layer and the depth of intestinal crypts in ΔF508*cftr*^−/−^ mouse small intestine. Scale bar: 100 μm. (**D**) Western blot analysis compares the expression of mature CFTR protein and α-smooth muscle actin (α-SMA) in WT (*n* = 7) and ΔF508*cftr*^−/−^ (*n* = 6) small intestine.

To evaluate the precise role of CFTR in intestinal inflammation, we set out to assess the immune cell infiltration, and histological manifestation in ΔF508*cftr*^−/−^ (ΔF508) mice compared to their corresponding wild-type littermates (WT). We first examined CFTR expression pattern using antibody targeting the N-terminus of CFTR in both WT and ΔF508 mouse intestine. As shown in Figure 1A, the expression of CFTR was mainly detected in the apical membrane of intestinal epithelial cells in WT mice. However, in ΔF508 mouse intestine, the expression level of CFTR was dramatically reduced and the mutated protein was mainly localized in the cytoplasm. To further evaluate the inflammatory status in the intestine, we compared the neutrophil and macrophage infiltration between WT and ΔF508 mice. Our results showed significantly increased neutrophil and macrophage infiltration in the mucosal layer of the small intestine of ΔF508 mice (Figure 1B). In addition, H&E staining of the small intestine showed significant increase of the thickness of smooth muscle layer and the depth of intestinal crypts in ΔF508 mice (Figure 1C). The changes in the smooth muscle layers of ΔF508 mouse intestine was further confirmed by the immunofluorescent staining and western blot analysis of smooth muscle marker α-smooth muscle actin (α-SMA) (Supplementary Figure S1 and Figure 1D). Taken together, these results validate the inflammatory phenotype in CF mouse intestine.

### Aberrant activation of NF-κB and suppression of β-catenin pathways in ΔF508 intestine

**Figure 2:**
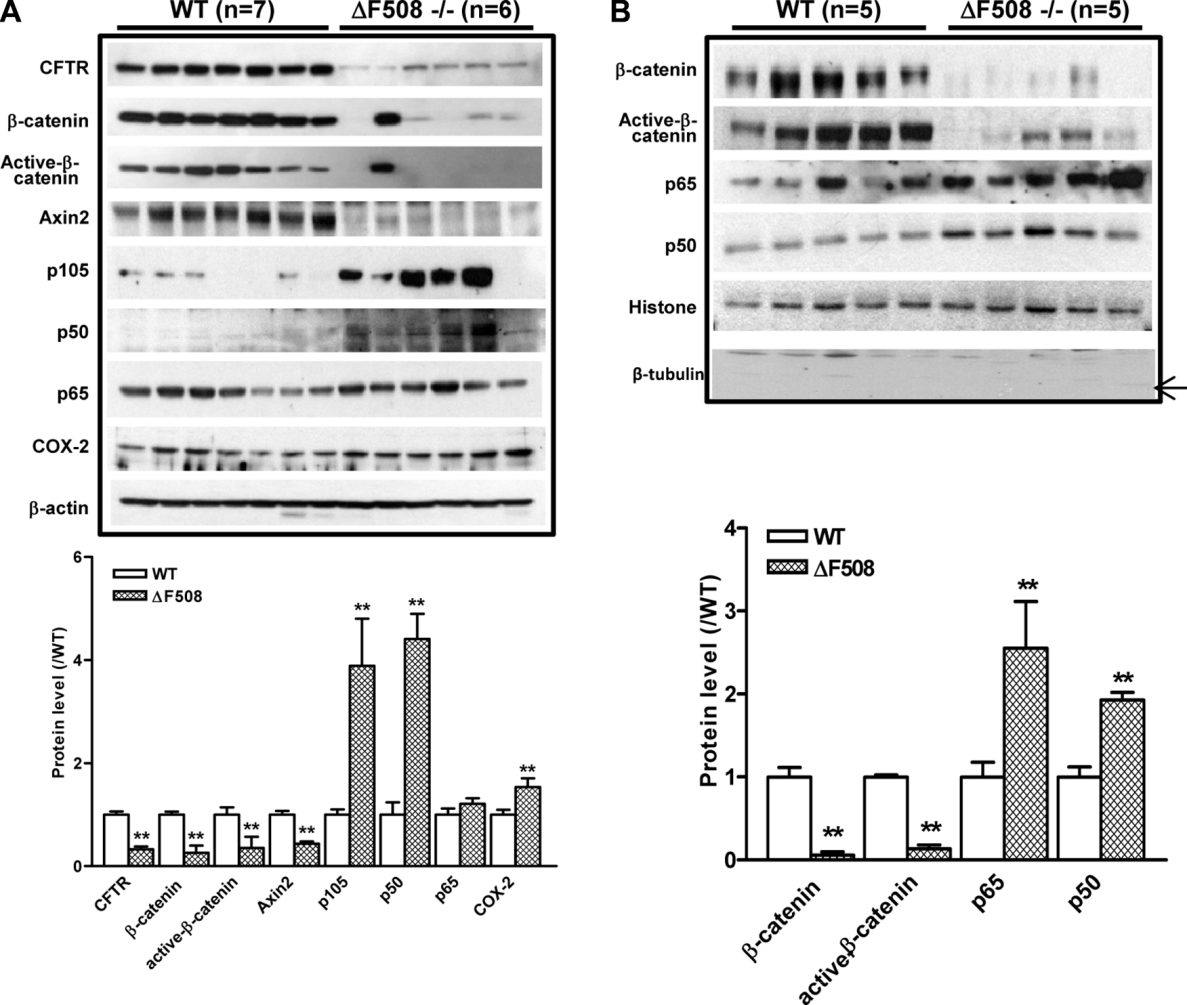
Aberrant activation of NF-κB and suppression of β-catenin pathways in ΔF508*cftr*^−/−^ mouse small intestine (**A**) Western blot of total lysates of WT (*n* = 7) and ΔF508*cftr*^−/−^ (*n* = 6) mouse small intestine shows reduced expression of β-catenin, active-β-catenin and Axin2; and upregulation of NF-κB p105, p50 as well as NF-κB downstream target COX-2 in ΔF508*cftr*^−/−^ mice. (**B**) Western blot of the nuclear fraction of WT (*n* = 5) and ΔF508*cftr*^−/−^ (*n* = 5) mouse small intestine shows significantly reduced nuclear β-catenin and active-β-catenin, whereas an increase of NF-κB p65 and p50 nuclear translocation in CF mice. The arrow indicates the expected band of tubulin. Image shown is the representative result from three independent experiments. (**p < 0.01, One-way ANOVA).

Since both NF-κB and β-catenin pathways have been implicated in the intestinal inflammation, we proceeded to examine the involvement of the two pathways in the upregulation of inflammatory phenotypes observed in the intestine of ΔF508 mice. We first determined the expression of NF-κB family members including p105, p65 and p50 in the total cell lysates derived from the small intestines of WT and ΔF508 mice. Our results showed that the expression of p105 and p50 was significantly upregulated in ΔF508 mouse intestine. In addition, the expression of COX-2, which can be transcriptionally activated by NF-κB, was also significantly upregulated. In contrast, the expression levels of β-catenin, active-β-catenin and β-catenin target protein Axin2 were reduced in the ΔF508 mouse intestine (Figure 2A). As the transcriptional activation of downstream targets of both NF-κB and β-catenin depends on their nuclear translocation, we further examined the expression of p65 and p50, total and active β-catenin in the nuclear fraction from WT and ΔF508 mouse intestine by western blot. The results showed that the expression levels of β-catenin and active-β-catenin were downregulated while that of p65 and p50 were upregulated in the nuclear fractions from ΔF508 mouse intestine compared to that derived from WT mice (Figure 2B). These results indicate that the NF-κB pathway is over-activated, whereas the β-catenin pathway is suppressed in the inflammatory intestine of ΔF508 mice.

### Knockdown of CFTR in intestinal epithelial cells leads to activation of NF-κB pathway and suppression of β-catenin pathway

**Figure 3:**
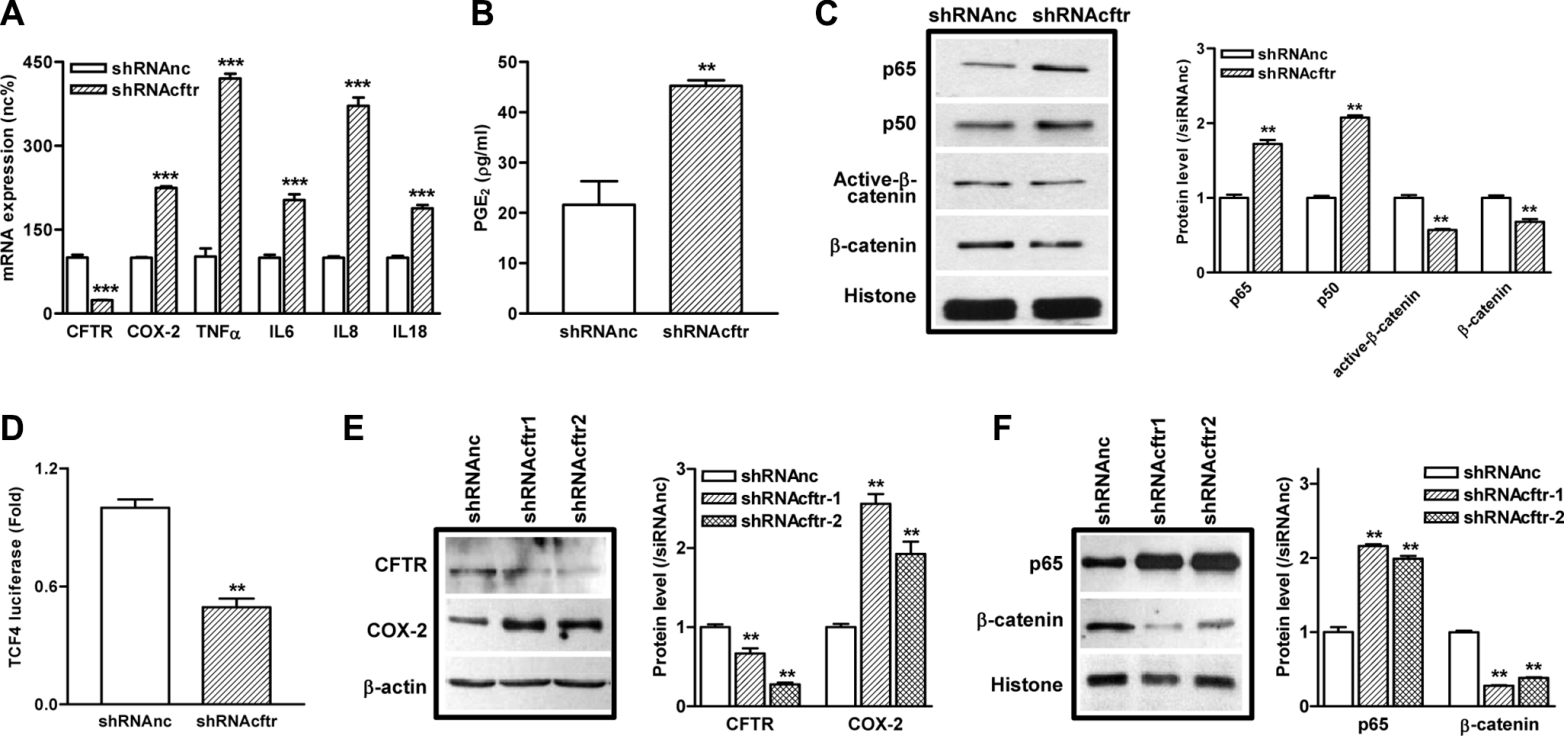
Knockdown of CFTR in intestinal epithelial cells leads to exaggerated inflammatory responses and suppression of β-catenin pathway (**A**) The mRNA levels of pro-inflammatory cytokines TNFα, IL6, IL8, and IL18 are upregulated after knock down of CFTR by shRNA in Caco-2 cells. (**B**) Knock down of CFTR increases PGE_2_ release in Caco-2 cells as determined by ELISA. (**C**) Activation of NF-κB pathway (p65 and p50) and repression of β-catenin and active-β-catenin in Caco-2 cells after CFTR knockdown as determined by western blot. (**D**) TCF-4 driven luciferase assay shows decreased TCF-4 transcription activity in Caco-2 cells after CFTR knockdown by shRNA. (**E**, **F**) In HRT-18 cells, knockdown of CFTR by shRNA increases COX-2 expression and nuclear translocation of NF-κB p65, whereas nucleus β-catenin is decreased as determined by western blot. Quantification data is acquired from three independent experiments. (**p < 0.01, ***p < 0.001, B and D, Student's *t*-test; A, C, E and F, One-way ANOVA).

Next, we used a highly enterocyte-like differentiated cell line Caco-2 as an *in vitro* model to further investigate the regulatory role of CFTR in the NF-κB-mediated inflammatory response. CFTR is highly expressed in Caco-2 cells (Supplementary Figure S2A), and Caco-2 has been shown to elicit inflammatory phenotype as stimulated by various extracellular factors [27, 28]. Our results showed that knockdown of CFTR in Caco-2 cells by shRNA significantly upregulated the mRNA expression of TNFα, IL6, IL8, and IL18, which have been well-characterized as pro-inflammatory cytokines in CF patients (Figure 3A). In corroboration with our previous findings in lung and prostate epithelial cells [20–22], knockdown of CFTR also increased the expression of COX-2 and the release of PGE_2_ (Figure 3A and 3B) in Caco-2 cells. These results indicate that suppression of CFTR in intestinal epithelial cells leads to the over-production of pro-inflammatory cytokines and mediators. Of interest, in consistent with the result from mouse intestine, suppression of CFTR significantly increased the expression of p65 and p50, whereas downregulated the expression of both β-catenin and active-β-catenin in the nucleus of Caco-2 cells (Figure 3C, Supplementary Figure S5). The expression of β-catenin, active-β-catenin and Axin2 was also significantly decreased in the total cell lysates of CFTR knock down Caco-2 cells (Supplementary Figure S3A). TCF4 is known to function as a co-transcription factor for β-catenin directed transcription [29]. To determine whether TCF4 signaling is involved, we used TCF4-driven luciferase assay and demonstrated that suppression of CFTR significantly decreased the transcriptional activity of TCF4 in Caco-2 cells, further confirming the repressive effect on β-catenin pathway by CFTR knock down (Figure 3D). The regulatory effect of CFTR suppression on the NF-κB and β-catenin pathways was further validated in another intestinal epithelial cell line HRT-18. As shown in Figure 3E and 3F, suppression of CFTR increased the expression of p65 and COX-2 whereas decreased the expression of β-catenin in HRT-18 cells.

### Activation of β-catenin inhibits NF-κB activity in ΔF508 mouse intestine and Caco-2 cells

**Figure 4:**
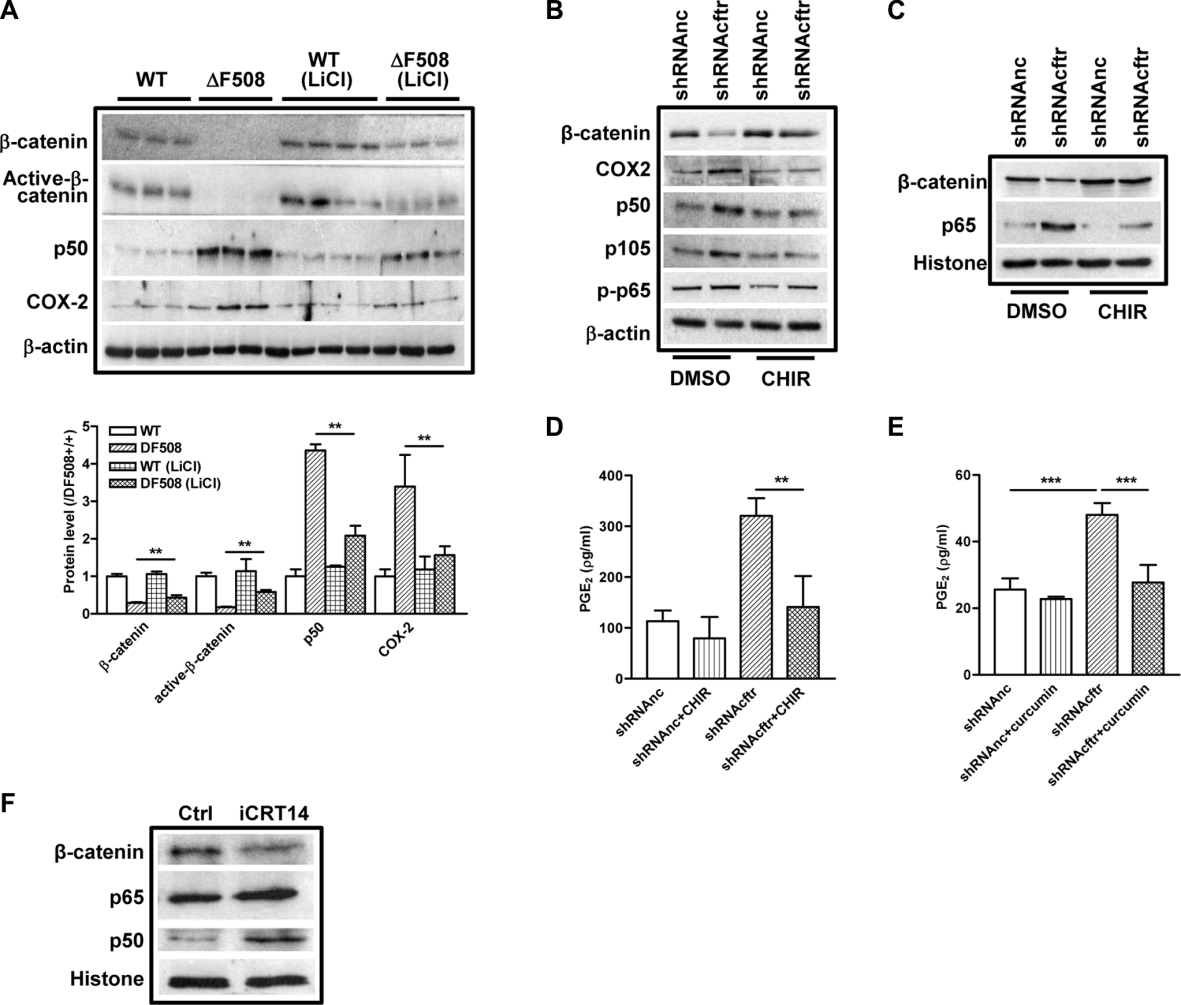
β-catenin inhibits NF-κB activity in ΔF508 *cftr*^−/−^ mouse intestine and Caco-2 cells (**A**) Wild type (*n* = 4)and ΔF508 *cftr*^−/−^ (*n* = 3) mice were treated with LiCl (200 mg/kg/day) for 9 days. Western blot shows partial recovery of total and active β-catenin expression in ΔF508 *cftr*^−/−^ mouse intestine. Increased expression levels of NF-κB p50 and COX-2 are reversed. (**B**) GSK3β inhibitor CHIR (10 μM) reverses CFTR knockdown-induced increase of p50, p105 and COX2 expression in Caco-2 cells, and restoration of β-catenin expression. (**C**) CHIR (10 μM) reverses CFTR knockdown-induced increase of nuclear translocation of p65 and reduction of β-catenin in Caco-2 cells. (**D**–**E**) Treatment with CHIR (10 μM) (E) or NF-κB inhibitor curcumin (30 μM) (E) for 24 hours reverse CFTR knockdown-induced increase of PGE_2_ release in Caco-2 cells. (**F**) Wnt/β-catenin pathway inhibitor iCRT14 (10 μM) increases nuclear translocation of NF-κB p65 and p50 in Caco-2 cells. Quantification data is acquired from three independent experiments. (**p < 0.01, ***p < 0.001 One-way ANOVA).

We have demonstrated that β-catenin pathway is downregulated whereas NF-κB pathway is activated in the ΔF508 mouse small intestine and CFTR knockdown intestinal epithelial cells. Given that β-catenin has been shown to suppress NF-κB activity [23, 24], we suspected that the over-activation of NF-κB-mediated inflammatory response in CF mouse intestine might be attributed to the suppression of β-catenin. To test this hypothesis, we treated the WT and ΔF508 mice with LiCl, a GSK3 inhibitor which activates β-catenin activity [30], and analyzed the alteration of NF-κB and COX-2 expression in mouse intestine by western blot. Our result showed that after LiCl treatment for 9 days, the expression levels of total β-catenin and active β-catenin in ΔF508 mouse intestine were partially recovered (about 50% of the WT controls), and the elevated expression levels of p50 and COX-2 were also significantly reduced (Figure 4A). In addition, the increased nuclear translocation of p65 was also reversed by LiCl treatment in ΔF508 mouse intestine (Supplementary Figure S3B). These results indicate that β-catenin suppresses NF-κB-COX-2 pathway in mouse intestine. To further elucidate the suppressive effect of β-catenin on NF-κB-mediated inflammatory response in intestinal epithelial cells, we treated control and CFTR knockdown-Caco-2 cells with 10 μM CHIR, another GSK3β inhibitor that activates β-catenin signaling [31], and examined the effect on NF-κB/COX-2/PGE_2_ pathway. Our western blot results showed that the elevated expression levels of p65, p50 and COX-2 in CFTR knockdown cells were significantly reduced by the CHIR treatment (Figure 4B and 4C). In addition, increased secretion of PGE_2_ in CFTR knockdown cells could be completely reversed by either CHIR or NF-κB inhibitor curcumin in Caco-2 cells (Figure 4D and 4E). To further establish the causative effect of β-catenin on NF-κB activation, we treated Caco-2 cells with β-catenin inhibitor iCRT14 and showed that suppression of β-catenin activity increased the nuclear translocation of p65 and p50 in Caco-2 cells (Figure 4F). Altogether, these results indicate that defect of CFTR activates NF-κB/COX-2/PGE_2_ pathway in intestine epithelial cells, which is attributed to the suppression of β-catenin pathway.

### CFTR interacts with β-catenin and prevents its degradation in Caco-2 cells and mouse small intestine

**Figure 5:**
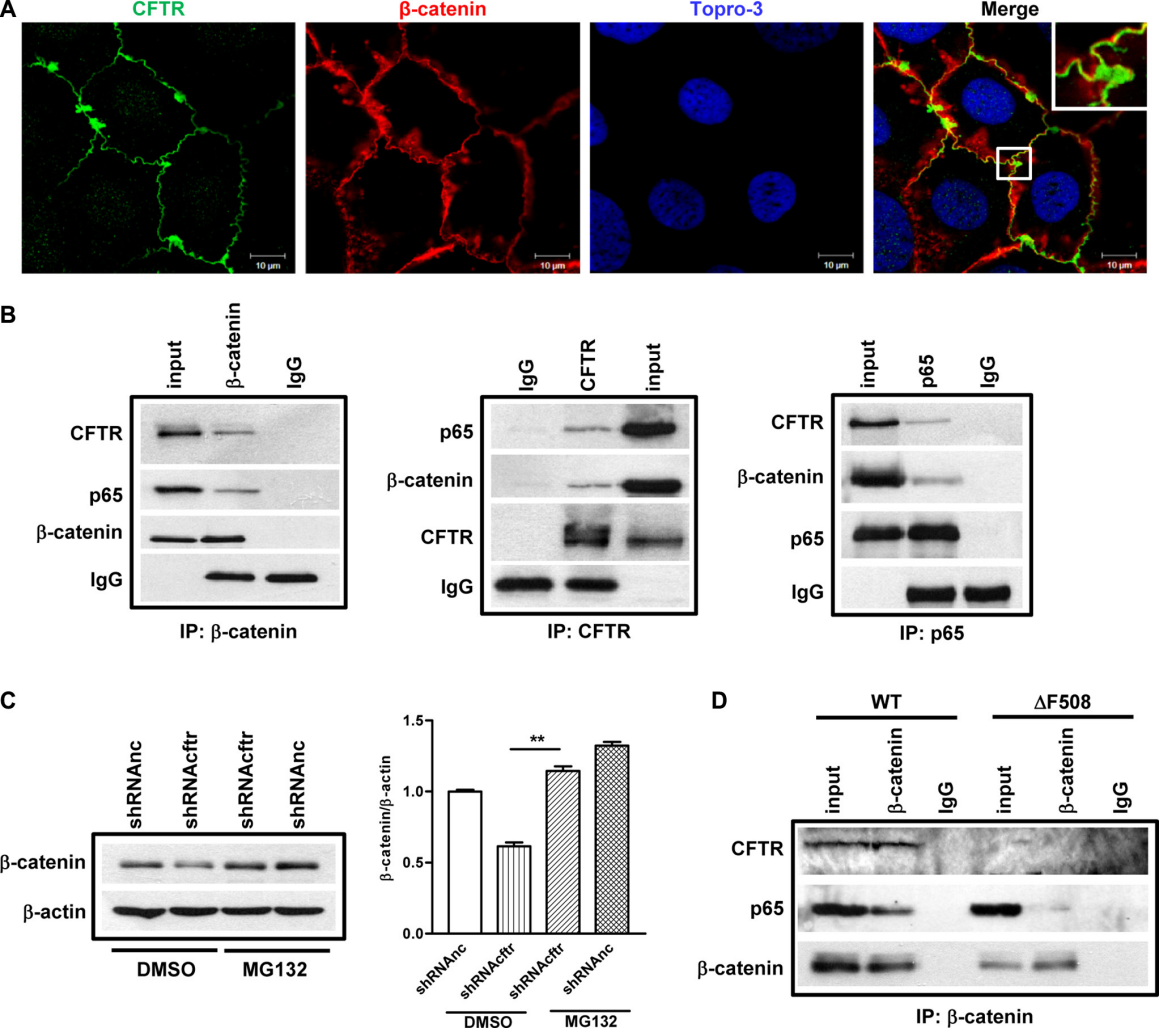
CFTR interacts with β-catenin and prevents its degradation in Caco-2 cells and mouse small intestine (**A**) Fluorescent immunostaining of CFTR (1:50, ACL-006, Alomone Labs) and β-catenin (1:200, CST2677, Cell Signaling) in Caco-2 cells shows co-localization of CFTR and β-catenin. Green color: CFTR; Red color: β-catenin; Blue color: To-pro3. Scal bar: 10 μm. (**B**) Co-immunoprecipitation study on Caco-2 cells. Left panel: both CFTR and NF-κB p65 immunoprecipitate with anti-β-catenin antibody; middle panel: β-catenin and NF-κB p65 immunoprecipitate with anti-CFTR antibody; right panel: CFTR and β-catenin immunoprecipitate with anti NF-κB p65 antibody. The blots shown are representative image from three independent experiments. (C) Proteasome inhibitor MG132 (10 μM) prevents CFTR knock down-induced β-catenin decrease in Caco-2 cells. Quantification data is acquired from three independent experiments. (D) Immunoprecipitation study in the intestine of wild type and ΔF508*cftr*^−/−^ mice. In wild type mouse intestine, both CFTR and NF-κB p65 are pulled down by anti-β-catenin antibody. In ΔF508*cftr*^−/−^ mouse intestine, ΔF508 CFTR protein is not co-immunoprecipitated with anti-β-catenin antibody, and the interaction between NF-κB p65 and β-catenin is significantly reduced (**p < 0.01, One-way ANOVA).

How does CFTR regulate β-catenin activity? Stabilization of β-catenin is of paramount importance for the subsequent nuclear translocation and activation of β-catenin pathway. On the other hand, emerging evidence has indicated that CFTR acts as a regulatory protein via interaction with a number of membrane and intracellular proteins [32]. Thus, we reasoned that CFTR might directly interact with β-catenin and prevent its degradation, and that mutation or downregulation of CFTR may lead to impaired interaction of CFTR with β-catenin resulting in enhanced β-catenin degradation. To investigate whether CFTR and β-catenin interact with each other, we first checked whether CFTR and β-catenin were co-localized in polarized Caco-2 cells. Results of confocal laser scanning microscopy showed the co-localization of CFTR and β-catenin at the cell-cell junctions in Caco-2 cells (Figure 5A). Then we used co-immunoprecipitation (co-IP) to further test if CFTR physically interacts with β-catenin in the cellular context. Caco-2 cell lysates were immunoprecipitated with anti-CFTR, β-catenin or NF-κB p65 antibodies together with protein A/G beads, and analyzed by Western blotting. As shown in Figure 5B, we found that both CFTR and p65 were detected in the immunoprecipitates of anti-β-catenin antibody, but not in the immunoprecipitates of IgG control (Figure 5B, left panel). Consistently, both β-catenin and p65 were detected in the immune-precipitates of anti-CFTR, whereas both CFTR and β-catenin were detected when pulling down with p65 antibody (Figure 5B, middle and right panels), suggesting that CFTR forms a complex with β-catenin and p65 in Caco-2 cells. We further tested the possibility that if the interaction between CFTR and β-catenin prevents β-catenin degradation. We treated the control and CFTR knockdown Caco-2 cells with proteasome inhibitor MG132, and found that MG132 completely rescued reduced β-catenin expression in CFTR knock down Caco-2 cells (Figure 5C). To further validate the interaction among CFTR, β-catenin and NF-κB *in vivo*, we conducted the co-IP experiment in WT and ΔF508 mouse intestine with anti-β-catenin antibody. Our results showed that β-catenin pulled down CFTR in WT but not ΔF508 intestine. In addition, the interaction between β-catenin and p65 was dramatically decreased in ΔF508 intestine compared to that in WT intestine, suggesting that defect of CFTR leads to reduced interaction between β-catenin and NF-κB.

## DISCUSSION

In the present study, we have revealed that over-activation of NF-κB-mediated inflammatory cascade leads to the inflammatory response in CF intestine. Moreover, we have provided the first hint to a link between CFTR and NF-κB via the β-catenin signaling pathway.

The inflammatory manifestations in CF intestine including infiltration of neutrophils and macrophage, thickness of smooth muscle layer, and increased depth of crypts (Figure 1A–1C). It should be noted that the expression of PCNA is dramatically increased in the intestinal crypts in ΔF508 mice compared to WT mice (data not shown), which is consistent with the recent paper showing that colon organoid formation is significantly increased in organoids created from cftr mutant mice compared with wild-type controls [33]. These findings may indicate a potential role of cftr in regulating the intestinal stem cell compartment during chronic inflammation. The effect of CFTR on inflammatory response has also been demonstrated in cell lines. Suppression of CFTR in Caco-2 cells increases the expression and secretion of multiple pro-inflammatory cytokines and mediators, such as TNFα, IL-8 and PGE_2_ (Figure 3A–3B), which have been detected in CF patient intestine [7]. Our results corroborate with other studies performed in various epithelial and non-epithelial cells indicating that deletion or mutations of CFTR confer a propensity to exaggerated inflammatory responses that are not related to bacterial infection [28, 34–36].

It has been previously reported that under LPS stimulation, defect of CFTR in the apical membrane of airway epithelial cells results in an excessive IκB phosphorylation, leading to destruction of IκB-NF-κB complex and NF-κB nuclear translocation. [37]. A recent study on intestinal Caco-2 cells also found that knock down of CFTR significantly increased IκB phosphorylation compared to control cells under IL-1β stimulation [28]. Interestingly, in both studies, the IκB phosphorylation levels in CFTR-knockdown cells were similar to that of their controls in basal conditions. These results indicate that increased IκB phosphorylation under stimulation cannot explain the activation of NF-κB and chronic inflammation status observed in CF even in the absence of infection. Despite the well-documented involvement of NF-κB activation in CF-related inflammations and well-demonstrated inverse relationship between CFTR and NF-κB activation in different experimental settings [37–41], the solid evidence linking CFTR mutation to aberrant NF-κB activation remains mysteriously missing. In the present study, we have shown that both CF mouse intestine and CFTR-knockdown intestinal epithelial cells exhibit consistent upregulation of NF-κB /COX-2/PGE_2_ pathway with concomitant downregulation of β-catenin and its signaling downstream targets (Figures 2 and 3). Importantly, inhibition of GSK-3β, a key component of the β-catenin degradation complex determining the fate of β-catenin, in CF mice and Caco-2 cells rescued β-catenin activity and suppressed the CFTR dysfuction-induced NF-κB/COX-2/PGE_2_ activity (Figure 4A–4E), suggesting that β-catenin may serve as an inhibitor of the NF-κB-mediated inflammatory response. This notion is further supported by our finding that treatment with β-catenin inhibitor enhanced the nuclear translocation of p65 and p50 in Caco-2 cells (Figure 4F). Taken together, our study strongly suggests the involvement of β-catenin downregulation as a key event in the activation of NF-κB/COX-2/PGE_2_ pathway in CF intestine.

**Figure 6:**
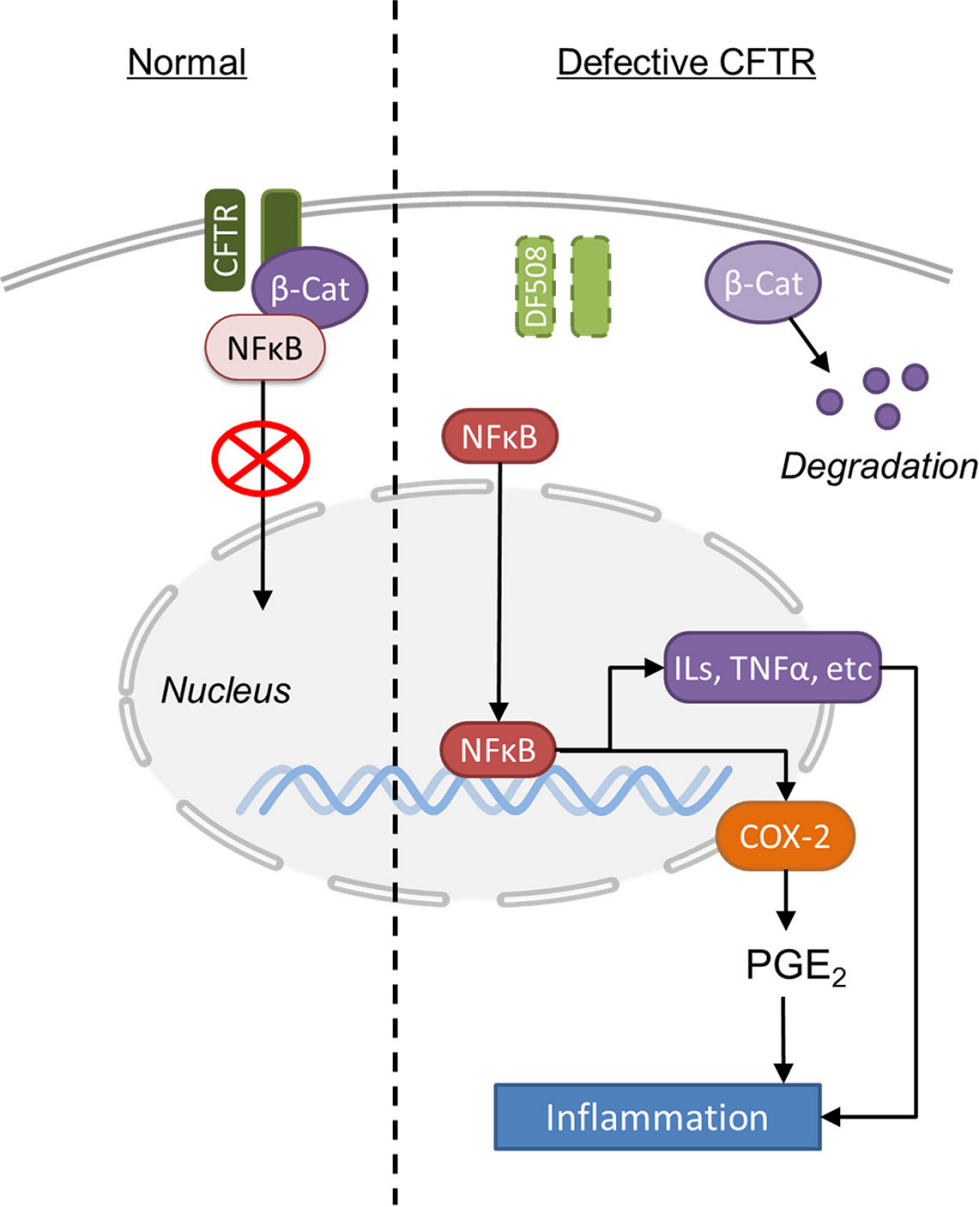
Schematic illustration of the molecular mechanisms underlying CFTR mutation-induced hyper-activation of NF-κB-Cox2-PGE_2_ pathway in mouse intestine

The most significant finding from our study is that CFTR forms a complex with β-catenin and NF-κB, which regulates NF-κB nuclear translocation and thus NF-κB-mediated inflammatory response (Figure 6). As a transcriptional activator of Wnt signaling, β-catenin plays a central role in intestinal development and homeostasis [42]. A critical control of this pathway is the degradation process of β-catenin [43]. Indeed, we have shown that CFTR and β-catenin co-localizes and physically interact with each other in Caco-2 and/or mouse intestine (Figure 5A–5C). CFTR mutation or downregulation reduces the nuclear translocation and activity of β-catenin (Figure 2 and Figure 3C–3E). Treatment with proteasome inhibitor completely reverses the reduced expression of β-catenin in Caco-2 cells (Figure 5C). These results indicate that CFTR stabilizes β-catenin and prevents it from degradation. More interestingly, the present study has further demonstrated that CFTR forms a complex with β-catenin and NF-κB in Caco-2 cells and mouse intestine. CFTR defect leads to decreased binding of β-catenin and NF-κB (Figure 5D and Supplementary Figure S4), which explains the augmentation of nuclear translocation of NF-κB seen in CF mouse intestine (Figure 2). Interestingly, previous studies have also demonstrated that β-catenin can stabilize NF-κB and inhibit the nuclear translocation of NF-κB through physical interaction, which is independent of the IκB-α in intestinal epithelial cells [23, 24]. The reduced interaction between β-catenin and NF-κB observed in CF mouse could be simply due to the accelerated degradation of β-catenin due to CFTR mutation. Interestingly, a recent comprehensive study on human lung epithelial cells has found that the CFTR interactome includes several hundred proteins, with β-catenin listed as a potential interacting protein [44]. Taken together, these results suggest that the interaction between β-catenin and NF-κB is subject to another level of regulation involving CFTR in the intestine, although whether the physical interaction among CFTR, β-catenin and NF-κB is direct or indirect awaits further investigation.

CF patients develop digestive tract cancers at a younger age and at a higher rate than non-CF individuals [45]. Chronic inflammation is considered to play a major role in the initiation, promotion and progression of colon cancer [46]. A latest study found that CFTR mutation increased intestinal tumor multiplicity in *Apc*^min^ mice. Although the mechanism was not clear, aberrant β-catenin expression was found in the intestine of *Apc*^min^*Cftr* knockout mice [47]. Thus, the presently demonstrated regulation of NF-κB activity by CFTR-β-catenin interaction may have implication in the development of GI cancer, as well as other chronic intestinal inflammatory disorders such as Crohn's disease and chronic inflammatory bowel diseases (IBD). Given the importance of Wnt/β-catenin pathway in many physiological and pathological conditions, the presently demonstrated critical role of CFTR in regulating β-catenin signaling provides novel insights into the physiological function of CFTR and pathogenesis of CF-related diseases in addition to the NF-κB-mediated intestinal inflammation seen in CF.

## MATERIALS AND METHODS

### Animals

B6.129s6-*Cftr*^tm1Kth^ (ΔF508) mice were purchased from Jackson's laboratory [48]. All animal experiments were conducted in accordance with the guidelines on animal experimentation of The Chinese University of Hong Kong and approved by the Animal Ethnics Committee of the University. Three months old *Cftr*^tm1Kth^ mice (wild type and ΔF508) were used in this project. Wild type and ΔF508 mice were assigned into vehicle control and LiCl treatment groups (*N* = 4). LiCl (200mg/kg, Sigma-Aldrich Co. Cat. L-0505) was dissolved in distill water and administrated by oral gavage for 8 days. At day 9, all mice were sacrificed by cervical dislocation. Intestine and colon were collected, stored in liquid nitrogen or fixed by 4% PFA for further use.

### Antibodies and reagents

Anti-C-terminal-CFTR (CFTR-C) was purchased from Alomone Labs (Jerusalem, Israel) and anti-N-terminal-CFTR (CFTR-N) was from Millipore (Billerica, MA, USA). Anti-GAPDH, anti-Histone H1, anti-β-tubulin, anti-PCNA, anti-COX2, anti-NFkB p105/50 and anti-p65 were obtained from Santa Cruz (Santa Cruz, CA, USA). Anti-α-Smooth Muscle Actin and anti-Axin 2 were obtained from Abcam (Cambridge, MA, USA). Anti-β-actin was purchased from Sigma (St. Louis, MO, USA). Anti-active-β-catenin and anti-β-catenin were obtained from cell signaling technology (Danvers, MA, USA). F4/80 antibody (clone Cl:A3-1) and Ly-6B.2 Alloantigen antibody (clone 7/4) specific for macrophage and neutrophils were purchased from Bio-Rad. CHIR was obtained from Merck Millipore. Lithium chloride was obtained from Sigma (St. Louis, MO, USA).

### Cell culture and lentiviral transduction

Human colon cancer cell lines Caco-2 and HRT-18 (ATCC) were cultured in MEM or RPMI-1640, respectively, supplemented with 10% FBS and 1% penicillin-streptomycin and maintained in 37^°^C and 5% CO_2_.

The lentiviral transduction particle encoding for shRNA against CFTR were purchased from Jima Inc. (shanghai, china). The sequences (5′ to 3′) of the two shRNA are GAA GTA GTG ATG GAG AAT GTA and TTG GAA AGG AGA CT AAC AAG T, respectively. These two shRNA duplexes are from two different regions of the human CFTR mRNA. The cells were plated in 24-well cell culture plates at a density of 10^4^ cells/well and incubated with 10 μl lentivirus (1 × 10^9^ TU/ml) for 8 hours. Viral vector containing noncoding shRNA were used as a control. Transduced cells were then expanded in 6-well cell culture plates, and finally transferred to 25 cm^2^ cell culture flasks. The transduced cells were selected in puromycin-containing (10 μg/ml) culture medium for two weeks.

### RNA extraction and RT-PCR

Total RNA of cells was extracted by TRIzol reagent (Invitrogen, Corporation, NY, USA) according to the manufacturer's instruction. 3–5 μg total RNA was used on reverse transcription reaction using moloney murine leukemia virus reverse transcriptase (M-MLV). The primer sequences are listed in Supplementary Table S1. PCR was carried out in triplicate on an Applied Biosystems PRISM PCR system (Life Technologies Corporation, California, USA). The conditions of PCR reaction were: 94°C 4 min, 94°C 30 s, 55°C 30 s, 72°C 30 s, repeat 35 cycles, and 72°C 10 min. The results were analyzed by sequence detection system software v2.2.1 (Life Technologies Corporation, California, USA). The amplified products were separated by 1% agarose gel.

### Real time quantitative PCR

The synthesized cDNA was used for Quantitative Real time PCR carried out on an Applied Biosystems 7500 Fast Real-Time PCR System. Taqman Gene xpression inventoried assay and taqman universial PCR master mix were used in the real time PCR reaction. The primer sequences are listed in Table 1. The cycling conditions were: stage1, 50°C for 2 min; stage 2, 95°C for 10 min; stage 3, 95°C for 15 sec, 60°C for 1 min. Stage 3 was repeated for 40 cycles. The samples are performed in triplicate and the transcriptional expression of gene of interest was indicated with average Ct normalized by Ct value of GAPDH.

### Western blotting

Cells were lysed in RIPA buffer (150 mM NaCl, 50 mM Tris–Cl, 1% NP-40, 0.5% DOC, 0.1% SDS, 1:100 PMSF, and 1:100 protein inhibitor) for 30 min on ice. The supernatant was collected as total protein after centrifugation at 12,000 rpm for 30 min. Equal amounts of protein were separated by SDS-PAGE western blotting. The protein bands were visualized by the enhanced chemiluminescence (ECL) assay (GE Health) following manufacturer's instructions and scanned by densitometer. Experiments were repeated for three times and the bands scanned and quantified. For nuclear protein extraction, after PBS washing cells was scraped in PBS and transferred to microtubes. The cell pellet was obtained by centrifuging for 5 min in room temperature at 2000 g. Supernatant was discarded and 50 μl low salt buffer A was added to cells. After vortex for 1min, the microtubes were incubated on ice for 5 min followed by centrifuging for 2 min in 4°C at 13000 rpm. The supernatant was collected carefully as cytoplasmic fraction and stored in −80°C. The nuclear pellet was resuspended with 25–30 μl high salt buffer B and rotates for 30 min in 4°C. The nuclear membrane fragments were removed by centrifuging at 13000 rpm for 10 min in 4°C. The supernatant was collected carefully as nuclear fraction and stored in −80°C.

### Immunohistochemistry and immunofluorescence staining

Fresh excised mouse small intestine was cut longitudinally and thoroughly washed by ice cold PBS, fixed in 4% paraformaldehyde at 4^°^C overnight, embedded in O.C.T. Compound (Sakura Finetek USA Inc.) and store in −80^°^C. 5 μm frozen sections (Cryotom^™^ FSE Cryostats, Thermo Scientifc) were mounted on Superfrost Plus slides (Fisher) and stored at −80^°^C for further immunostaining or hematoxylin and eosin staining. For Immunofluorescence staining, frozen sections were incubated in retrieval buffer (sodium citrate, pH 6.0, at 95^°^C) for 15 min. After blocking with 5% normal donkey serum 1 hour at room temperature, sections were incubated with primary antibodies overnight at 4°C in a humidified chamber. Control sections were incubated in the absence of primary antibodies. After PBS wash three times, the sections were then incubated with fluorescent secondary antibody (Alexa Fluor 488 (green) or 568 (red) (1:500, Thermo Fisher Scientific) for 60 min at room temperature. Sections were counterstained by Topro-3 (0.1 μM) or DAPI (5 μg/ml) 15 minutes for nuclear staining. At last, the sections were mounted with cover slip and visualized under confocal laser scanning microscopy (LSM 520, Zeiss Inc).

Caco-2 cells were grown on glass coverslips coated with collagen for 3–10 days before being fixed in cold methanol for 10 minutes. Fixed Caco-2 cells were blocked in 1% bovine serum albumin (BSA) for 1 hour at room temperature and then incubated with primary antibodies overnight at 4°C. After three times wash in PBS, sections were incubated with fluorescent secondary antibodies for 1 h at room temperature. The nucleus was counterstained with To-pro 3. Cells were then visualized under Confocal laser scanning microscopy (LSM 520, Zeiss Inc).

For immunohistochemistry staining of macrophage and neutrophil in mouse intestine, a ABC kit (VectaStain elite ABC kit, PK6100, VectorLaboratories) was used and staining protocol was followed according to manufactory's instruction. In brief, Mouse intestine frozen sections were rinsed 5 min in PBS, quenching of endogenous peroxidase activity by incubation the sections in 3% H_2_O_2_ for 10 minutes, blocking in 2% normal donkey serum for 30 minutes at room temperature, incubating with primary antibodies F4/80 and Ly-6B.2 at 1:50 dilution at 4^°^C overnight, after 3 washes in PBS, incubating with biotinylated secondary antibody for 30 minutes, then the sections were developed in peroxidase substrate solution and counterstained with hematoxylin, cleared and mounted for assessment under microscope.

### Co-immunoprecipitation assay

Cells were allowed to grow in 75 cm^2^ flask till confluency. The cells were lysed with ice-cold lysis buffer (50 mM HEPES, pH 8.3, 420 mM KCl, 0.1% NP-40, 1 mM EDTA) for 30 min on ice. Supernatant was collected after centrifugation at 14,000 rpm at 4°C for 15 min. 100 μl supernatant was saved as input and stored at −80°C. The spin-down cells were incubated with 5 μg anti-N-terminal-CFTR, NF-kb or β-catenin antibodies and normal mouse/rabbit IgG together with Protein A/G (Santa Cruz, CA, USA) at 4°C overnight with rotation. The beads were harvested by centrifugation on the following day and washed 5 times with PBS. Sample loading buffer was added to the beads and incubated for 30 min at room temperature. The results were analyzed by Western blot.

### PGE2 ELISA assay

For the conditional medium collection, cells were seed in 6 well plate with 3 ml full medium. 48 h later, mediam wascollected and centrifuged at 3000 rpm for 3 min to remove cell fragment, supernatant was collected and stored at −80°C for further use. PGE2 ELISA assay (Cayman Chemical Inc. Cat. 514010) was performed according to manufactory's protocol.

### TCF4/β-catenin dual-luciferase reporter assay

The plasmid pcDNA3.1-TCF4 was transiently transfected to Caco-2 cells (control group and CFTR knockdown group) cultured in 6 well plate at 80% confluent by Lipofectamine 2000 according to manufactory's protocol. Renilla-Luc (0.005 μg/well) was added as an internal control. Firefly and renilla luciferase activity was assessed by respective assay kit (Promega Inc.). The relative luciferase activity was defined as the ratio of readout for firefly luciferase to that for renilla luciferase with that of control group set as 1.0.

### Statistical analysis

Data were input into Prism (GraphPad Software. Inc.) and expressed as mean ± SEM. Student's *t* test was used for comparison of two groups, and One-way ANOVA was used for comparison of three or more groups. Results were considered statistically significant at *P* < 0.05.

## SUPPLEMENTARY MATERIALS FIGURES AND TABLES


