# Supplementary material for: Defective CFTR- β-catenin interaction promotes NF-κB nuclear translocation and intestinal inflammation in cystic fibrosis
Source: Oncotarget. 2016 Aug 31;7(39):64030–42. doi: 10.18632/oncotarget.11747 (PMC5325423; doi:10.18632/oncotarget.11747)
Supplement: Supplementary file 1 [file oncotarget-07-64030-s001.pdf]

Defective CFTR- β-catenin interaction promotes NF-κB nuclear translocation and intestinal inflammation in cystic fibrosis

Supplementary Materials

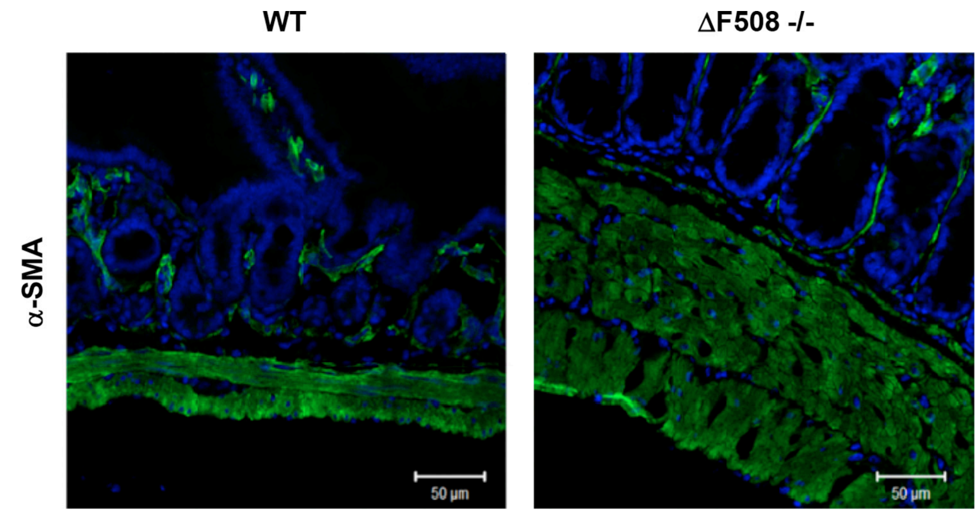

Supplementary Figure S1: Fluorescent immunostaining of α-SMA shows significant increase of the thickness of smooth muscle layer in ΔF508 mouse intestine. Green color: α-SMA (anti-alpha smooth muscle actin, 1:100, ab5694, Abcam); blue color: DAPI; scale bar: 50 µm).

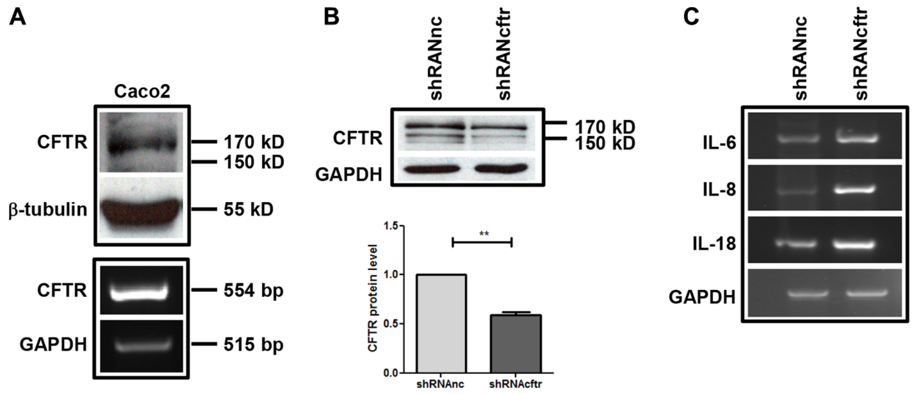

Supplementary Figure S2: (**A**) CFTR is endogenously expressed in Caco-2 cells. upper panel: western blot of CFTR and tubulin in Caco-2 cells; lower panel: RT-PCR results show CFTR mRNA expression in Caco-2 cells. (**B**) Western blot shows CFTR knock down by transfection of shRNA in Caco-2 cells. Quantification data was resulted from three independent experiments (**C**) RT-PCR results show increased mRNA levels of IL6, IL8 and IL18 in Caco-2 cells with CFTR knockdown.

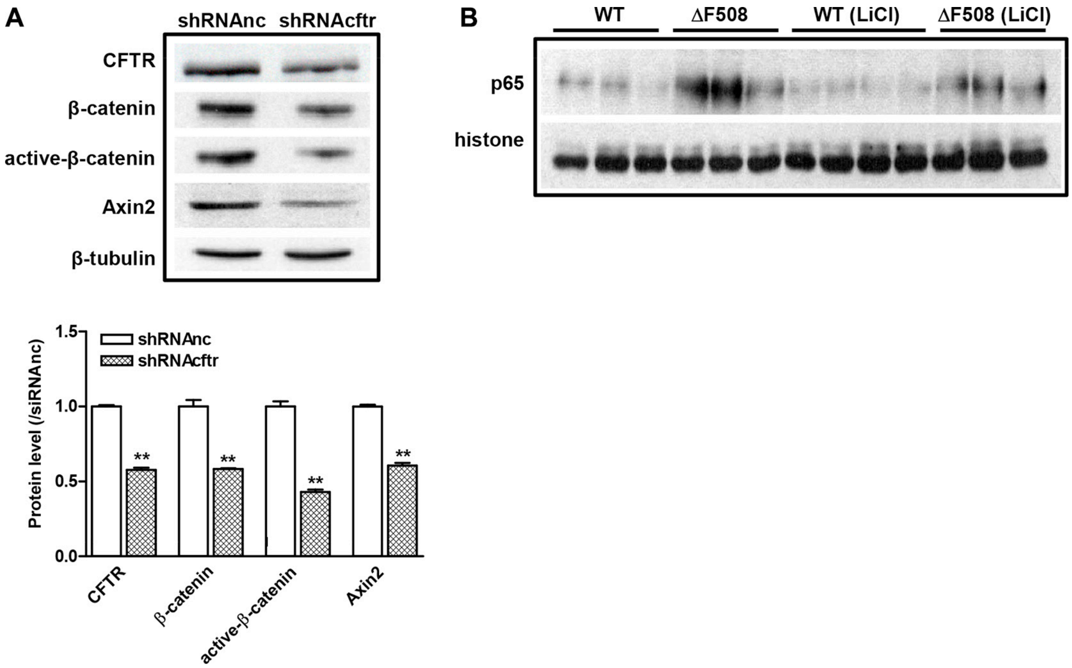

Supplementary Figure S3: (**A**) Upper panel: western blot shows down regulation of β-catenin, active-β-catenin and Axin2 in Caco-2 cells after CFTR knockdown. Lower panel: bar chart shows semi-quantitative analysis of western results of β-catenin, active-β-catenin and Axin2 in CFTR knock down Caco-2 cells. (**B**) LiCl treatment reverses the increase of NFκB p65 nucleus translocation in DF508 mouse intestine. WT or DF508 mice are treated with LiCl (200 mg/kg/day) for 9 days. Nucleus fraction of mouse small intestine is extracted. Western blot shows increased NFκB p65 nucleus translocation in DF508 mouse intestine, which can be reversed by LiCl treatment.

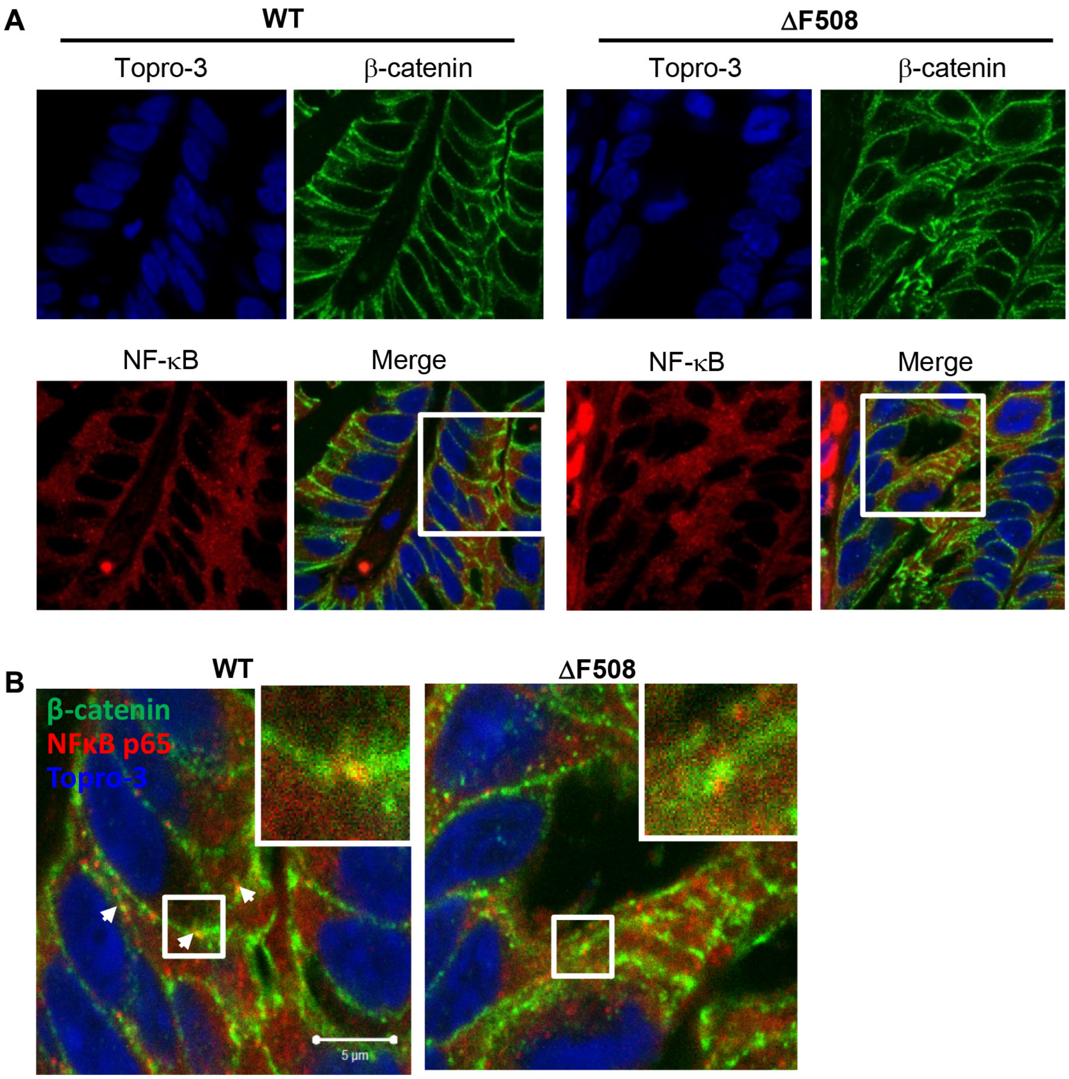

Supplementary Figure S4: (**A**) Immunofluorescence staining of β-catenin and NF-κB in WT and ΔF508*cftr*$^{-/-}$ mouse small intestine, scale bar = 10 µM (**B**) β-catenin interacts with NF-κB in WT but not ΔF508*cftr*$^{-/-}$ mouse intestine. Squared area captured at A is enlarged in B. scale bar: 5 µm.

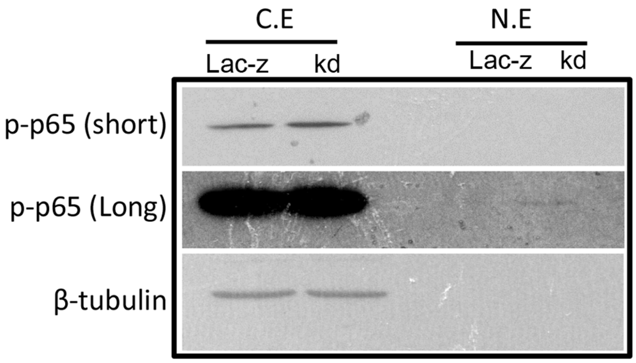

Supplementary Figure S5: The expression of phospho-p65 (Ser 536) in control and CFTR knockdown cells.

Supplementary Table S1: Primer lists

Name	sequence	product
CFTR_F	GTGTGATTCCACCTTCTCCAA	149 bp
CFTR_R	GCCTGGCACCATTAAAGAAA	149bp
COX2_F	CCCTTGGGTGTCAAAGGTAA	143 bp
COX2_R	AACTGATGCGTGAAGTGCTG	143 bp
TNFα_F	TCAGCCTCTTCTCCTTCCTG	124 bp
TNFα_R	GCCAGAGGGCTGATTAGAGA	124 bp
IL6_F	TTCAATGAGGAGACTTGCCTG	349 bp
IL6_R	ACAACAACAATCTGAGGTGCC	349 bp
IL8_F	TCT CTT GGC AGC CTT CCT G	331 bp
IL8_R	GAA GTT TCA CTG GCA TCT TCA C	331 bp
IL18_F	GCT TGA ATC TAA ATT ATC AGT C	335 bp
IL18_R	CAA ATT GCA TCT TAT TAT CAT G	335 bp
GAPDH_F	AGGGTCATCATCTCTGCC	245 bp
GAPDH_R	CCATCACGCCACAGTTTC	245 bp
CFTR_F	AGCTGGACCAGACCAATTTTGAGGAAA	554 bp
CFTR_R	CCACACGAAATGTGCCAATGCAAGTCC	554 bp
GAPDH	TCC CAT CAC CAT CTT CCA G	515 bp
GAPDH	TCC ACC ACT GAC ACG TTG	515 bp
